# Effect of Thickness and Bonding Technique on Fatigue and Fracture Resistance of Feldspathic Ultra-Thin Laminate Veneers

**DOI:** 10.1055/s-0042-1745770

**Published:** 2022-06-21

**Authors:** Amna Mohamed Ahmed Hassan Al-Ali, Nadia Khalifa, Amir Hadj-Hamou, Soumya Sheela, Hatem M. El-Damanhoury

**Affiliations:** 1 Department of Preventive and Restorative Dentistry, College of Dental Medicine, University of Sharjah, Sharjah, United Arab Emirates; 2 Dental Biomaterials Research Laboratory, Research Institute for Medical & Health Sciences, University of Sharjah, Sharjah, United Arab Emirates

**Keywords:** fatigue resistance, fracture resistance, laminate veneers, preheated composite, resin cement

## Abstract

**Objectives** To evaluate the fatigue and fracture resistance of ultra-thin laminate veneers (UTLV) with two different thicknesses and two different bonding protocols.

**Materials and Methods** A total of 64 flat enamel surfaces were assigned to either 0.2 or 0.4 mm UTLV. The UTLV were further subdivided and assigned to one of two bonding techniques: adhesive resin cement(RC( or preheated restorative resin composite (HC) ( *n* = 16). Eight samples were fatigued with 750,000 mechanical cycles and 8,000 thermal cycles between 5 and 55°C in a chewing simulator, and the failure mode was evaluated using a stereomicroscope and SEM. The other eight samples from each group were loaded to failure in a universal testing machine to test the fracture resistance. Fisher's exact Probability test was used to analyze the fatigue test results, and two-way analysis of variance and Bonferroni's test were used to analyze the fracture resistance test results.

**Results** The difference in fatigue resistance between failure proportions in different groups was statistically different ( *p* < 0.05). The 0.4-mm-thick UTLV had similar results regardless of the bonding technique, while 0.2-mm-thick UTLV only showed comparable results when cemented with preheated HC. No statistically significant difference was found in fracture resistance between the tested groups ( *p* > 0.05).

**Conclusion** The Bonding technique and the thickness of the UTLV had impacted fatigue resistance but had no significant effect on the fracture resistance. bonding of UTLV with preheated composite increases their fatigue resistance. Different testing approaches delivered different results.

## Introduction

Dental esthetics is a booming industry propelled by the desire for perfect teeth and a beautiful smile. Because of ongoing advancements in dental ceramics and bonding techniques, porcelain veneers are in high demand. Laminate veneers are wafer-thin shells ∼0.5 to 1.0 mm thick, made from dental ceramics that are bonded onto the facial surface of the teeth. 1 These are usually fabricated from different types of dental ceramics. 2 Glass feldspathic porcelain is derived from the naturally occurring feldspar and was the first dental ceramic used for esthetic veneers. 3 Other forms of synthetic glass ceramics are leucite-based, lithium disilicate, and fluorapatite-based ceramics. 4 Although they have more favorable mechanical properties, the strength of conventional feldspathic porcelain is commonly sufficient for anterior porcelain veneers with more esthetic natural color and translucency. 5 6 A study reported that porcelain laminate veneers had a very high 10-year survival rate (95%) when bonded to enamel substrate. 7 In contrast, non-feldspathic veneers, such as Empress veneers, tend to have a 5-year survival rate of 92.4% and a 10-year survival rate of 66 to 94%. 8 9

Porcelain veneers should ideally preserve dental tissue and avoid traumatizing the surrounding soft tissue. 10 Laminate veneers have superior optical reflectance and may have improved gingival tissue response when the margins are placed supragingivally. 11 The minimally invasive preparation is a new approach, in which preparation must be restricted to enamel in terms of the margin, and with a preparation depth of ∼0.2 to 0.3 mm. Cementation of ceramic veneers mainly depends on resin bonding to enamel, which was reported to strengthen the ultra-thin laminate veneers (UTLV). 12 This minimal preparation design guarantees better bonding to enamel and to avoid the probability for postoperative sensitivity, which may happen if the preparation is extended to dentin. 13

The conventional technique used for adhesion is the resin cement (RC) bonding methods, which increases the fracture resistance of porcelain veneers. 14 An alternative cementation technique was reported by Rickman et al., using preheated resin composite (HC) filling material. Heating the HC restoration reduces the polymerization shrinkage and increases fatigue resistance at the margins of the veneer much more in comparison to the conventional unfilled resin luting cement. 15 Friedman demonstrated the effectiveness of porcelain veneers bonded with a microhybrid restorative HC for over 15 years. 16 Another study by Gresnigt et al. investigated the use of preheated restorative HC resistance as a luting agent for the cementation of laminate veneers, and reported considerably superior survival rate and fracture resistance. 17

The *in vitro* research is crucial for evaluating the mechanical properties of dental materials for implementation and survival in the oral cavity. 18 There are limited data in the published literature regarding the use of UTLV and the effect of different bonding techniques on their mechanical behavior. Therefore, this study was conducted to investigate the fatigue and fracture resistance of UTLV with two different thicknesses, cemented with either adhesive RC or preheated restorative HC. The null hypothesis is that there is no difference in fatigue and fracture resistance of UTLV with different thicknesses and bonding procedures. The second null hypothesis is that the two testing approaches used in this study would not deliver different results.

## Materials and Methods

This experimental study was conducted on 64 sound human maxillary central incisors, collected from the University Dental Hospital after gaining approval from the University of Sharjah Research Ethics Committee (reference number: REC-20-06-25-03-S), and obtaining patients' informed consent. The teeth were cleaned and stored in distilled water in a refrigerator until being tested. Later, all the teeth were inspected under the stereomicroscope for the presence of cracks, decay, or previous restorations.

Teeth were divided randomly into two groups, 32 teeth each, and assigned to either 0.2-mm-thick veneers or 0.4-mm-thick veneers. Each group was further subdivided into two subgroups, 16 samples each, according to the material used for cementation. In the first subgroup, the UTLV was cemented with conventional resin cement RC Conventional (groups RC-0.2 and RC-0.4), and the second subgroup was cemented with preheated resin composite HC (groups HC-0.2 and HC-0.4). A power and sample size calculation software (PS, Version 3.1.2, Vanderbilt University, Nashville, Tennessee, United States) was used to calculate the sample size based on the pilot study for 90% power at α = 0.05. The materials used in the research experiments: brands, types, chemical compositions, manufacturers, and lot numbers are listed in Table 1 .

**Table 1:** Material applications, brands, types, chemical compositions, manufacturers, and lot numbers used in the experiments

**Brand**	**Manufacturer**	**Application**	**Chemical composition**	**Fillers wt%**	**Lot no.**
Vita Blocks Mark II	VITA Zahnfabrik, Bad Säckingen, Germany	Machinable ceramics	Feldspar ceramic	–	D-79713
N-Etch	Ivoclar Vivadent, Schaan, Liechtenstein	Tooth etching agent	37% Phosphoric acid	–	Y39063
Tetric N-bond universal	Ivoclar Vivadent, Schaan, Liechtenstein	Light-curing single-component dental adhesive	Methacrylates, ethanol, water, highly dispersed silicon dioxide, initiator, and stabilizers	–	Z002G4
Ceram-Etch	iTENA, Villepinte, France	Ceramic etching gel	9% Buffered hydrofluoric acid gel	–	4187–35PFXE
Monobond N	Ivoclar Vivadent, Schaan, Liechtenstein	Universal primer	Silane methacrylate, phosphoric acid methacrylate, and sulfide methacrylate	–	Y48877
Variolink Esthetic LC	Ivoclar Vivadent, Schaan, Liechtenstein	Light-curing resin-based dental luting material	Urethane dimethacrylate, methacrylate monomers. Ytterbium trifluoride and spheroid mixed oxide inorganic fillers with 0.04–0.2 μm particle size are initiators, stabilizers, and pigments	38 wt%	Y38575
IPS Empress Direct	Ivoclar Vivadent, Schaan, Liechtenstein	Light-curing resin-based dental restorative material	Dimethacrylates, barium glass filler, mixed oxide, Ba-Al-fluorosilicate glass, copolymer, initiators, stabilizers, and pigments	79.6 wt%	W93802

The tested UTLV were fabricated from monochromatic, feldspar computer-aided design/computer-aided manufacturing blocks (Vitablocs Mark II, blanks size I-12, VITA Zahnfabrik, Bad Säckingen, Germany) with a dimensions of 12 × 10 × 15 mm. Each block was sectioned longitudinally from the center, and then rotated 90 degrees and sliced into sections with 6 mm width and 10 mm length and either 0.2 and 0.4 mm thickness, using a precision sectioning saw (Isomet 1000, Buehler, Ratingen, Germany). The thickness of each slice was measured using a digital caliper with a resolution of 0.01 mm (Reichelt Elektronik GmbH & Co., Sande, Germany). The laminate veneers were then inspected under the stereomicroscope to ensure the absence of any cracks induced during sectioning. One surface of the veneer was marked with a small dot by a permanent marker as the untreated surface, and the veneer slice was held by an applicator stick with an adhesive tip (OptraStick, Ivoclar Vivadent, Schaan, Liechtenstien) for handling during treatment and to simplify the cementation procedure. The other surface of the veneer was etched using 9% hydrofluoric acid gel for 60 seconds, followed by cleaning with an air/water spray for 30 seconds. A silane primer was applied to the porcelain surface for 60 seconds and then air-dried.

The labial surfaces of the teeth underwent flattening and roughening using 600 grit sandpaper discs mounted on an automatic polisher (Forcipol 2V Grinder and Polisher, Kemet International Ltd, Kent, UK). Each tooth was stabilized by a green stick compound in a cubicle-shaped resin block. Prepared enamel surfaces were etched with a 37% phosphoric acid etching (N-etch, Ivoclar Vivadent) for 30 seconds, rinsed and dried thoroughly with oil-free air/water spray for 30 seconds. A light-cured bonding system (Tetric N-bond universal, Ivoclar Vivadent) was applied for 20 seconds using a microbrush and air-thinned for 5 seconds and light-cured for 20 seconds using a light-curing unit (LCU) (MiniLED, Satelec, Mérignac, France) with 1,250 mW/cm ^2^ light intensity output. For groups RC-0.2 and RC-0.4, a light-cured RC (Variolink Esthetic LC, Ivoclar Vivadent) was used for cementation, while in groups HC-0.2 and HC-0.4, the ceramic veneers were cemented with restorative HC (IPS Empress Direct, A2 Enamel, Ivoclar Vivadent), in which the composite syringe was preheated at 68°C using a composite warmer device (Calset Composite Warmer, AdDent Inc., Connecticut, United States) for 3 minutes along with heating the plastic instrument simultaneously. The veneers were placed on the flattened enamel surface, and the edge of the veneer was aligned with the incisal edge of the tooth. Cementation of the UTLV to the tooth surface was done under a 50-g standardized load applied for 60 seconds after removing the applicator stick. A microbrush was used to remove any excessive luting material carefully. Afterward, a glycerin-based gel (Liquid Strip, Ivoclar Vivadent) was applied on the margins to prevent the oxygen inhibition of polymerization during light curing. All the samples' facial and incisal surfaces were light-cured for 20 seconds. The light intensity of the LCU was checked every five samples using the built-in radiometer in the LCU. The samples were kept in distilled water in a 37°C incubator for 24 hours, then finishing was done using sandpaper discs (Sof-Lex, 3M Oral care, St Paul, Minnesota, United States) to remove the overhanging margins of the veneers.

### Fatigue Resistance Testing

The samples were positioned vertically along the long axis of the tooth in the middle of a Teflon holder with a surveyor. The holder was filled with self-cure polymethylmethacrylate up to 2 mm from the cementoenamel junction. After the resin set, all the samples were stored in distilled water for 24 hours at 37°C before testing. Eight specimens from each experimental group were placed in an eight-chamber chewing simulator (Chewing Simulator CS-8, SD Mechatronik, Feldkirchen-Westerham, Germany) with two stepper motors that produce vertical (6mm) and horizontal (2mm) movements controlled by a computer. The samples were subjected to 750,000 mechanical cycles of 40 N load with 1.6 Hz frequency. The load was applied at the middle third of the incisal surface through a ball-shaped steatite antagonist with a 6-mm diameter. Samples were simultaneously subjected to 8,000 thermal cycles between 5 and 55°C in distilled water with 30 seconds dwell time in each temperature extreme.

Following the fatigue testing, the failure mode of the samples was evaluated under ×40 magnification using an optical stereomicroscope (Wild M3Z, Leica Microsystems GmbH, Wetzlar, Germany). The samples were classified into Failure (cracked or fractured) or no-Failure. Additionally, three representative specimens from each group were sputter-coated with a layer of gold (80%)/palladium (20%) and analyzed using a cold field emission scanning electron microscope (SEM) (VEGA3, Tescan, Brno, Czech Republic). Images were taken at 10 kV at a magnification of 2.5 kx

### Fracture Resistance Testing

The specimens were placed vertically in the middle of a Teflon holder with a surveyor along the long axis of the UTLV slice. The holder was filled with stone up to 2 mm from the cementoenamel junction. All the samples were stored in distilled water for 24 hours at 37°C before testing; the remaining eight samples from each group ( *n* = 8) were evaluated for fracture resistance in a universal testing machine (multiparameter testing machine 5ST, Tinius Olsen, Surrey, UK). The samples were held with a special jig to maintain the vertical orientation during testing, and a load was directly applied to the incisal edge of the veneers using a flat plunger (1 mm width and 10 mm length) attached to a 5-kg load cell, running at a crosshead speed of 1 mm/min. The maximum force at failure was automatically recorded in Newton (N) using the data acquisition and machine control system software (Horizon, Tinius Olsen, Surrey, UK).

### Statistical Analysis

Data were collected, tabulated, and statistically analyzed using SPSS software (SPSS, Version 20, IBM, Armonk, New York, United States). Shapiro–Wilk's test was used to test the normality of all the quantitative variables for further choice of appropriate parametric or nonparametric tests. Results of the sample examination after the fatigue test were analyzed using Fisher's exact Probability test to assess differences between the groups. For the fracture resistance test results, all the variables were normally distributed allowing the use of parametric tests. The general linear model (GLM) with the two-way analysis of variance (ANOVA) analyzes the two main factors with their interaction. The pair-wise comparison was done using the Bonferroni method. The significance level was considered at *p* < 0.05, while *p* < 0.01 was considered highly significant.

## Results

**Fig. 1:**
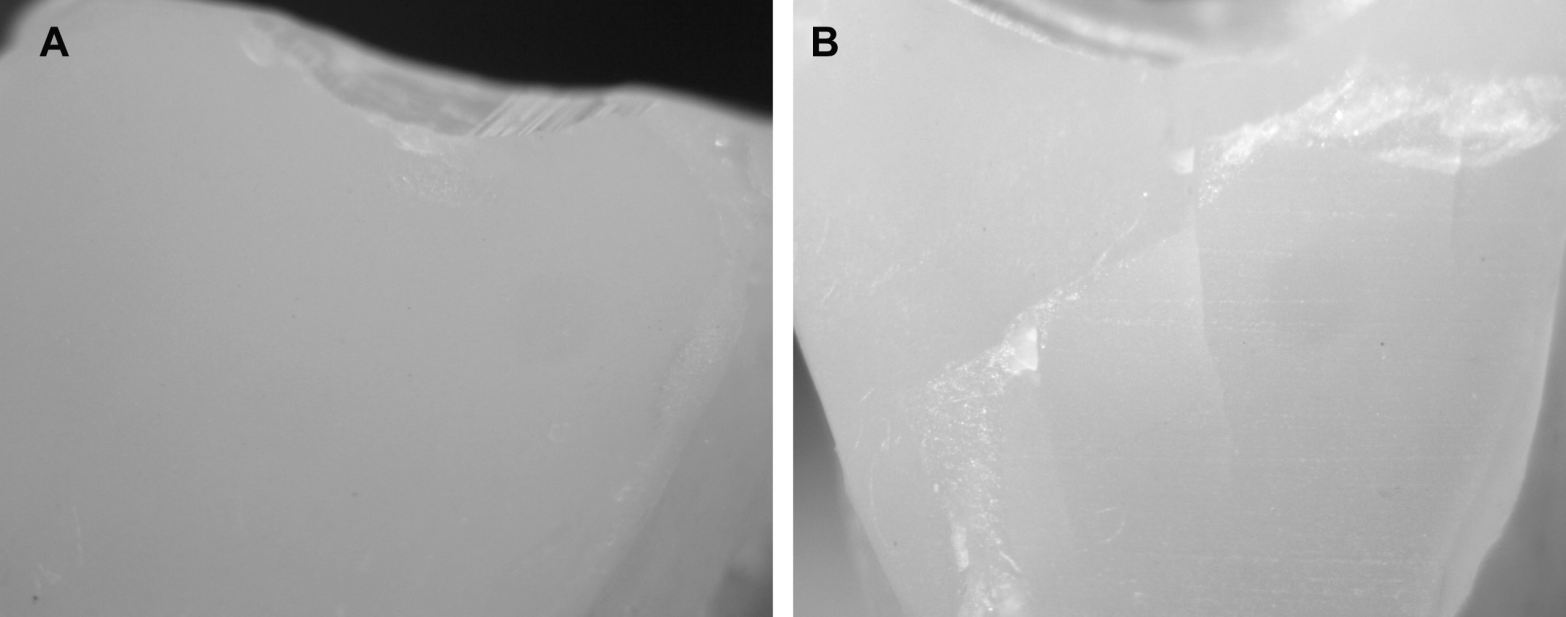
A stereomicroscopic images (×40 magnification) showing failure of the ultra-thin laminate veneers after thermomechanical fatigue; ( **A** ) marginal chipping and ( **B** ) cracking and fracture.

**Fig. 2:**
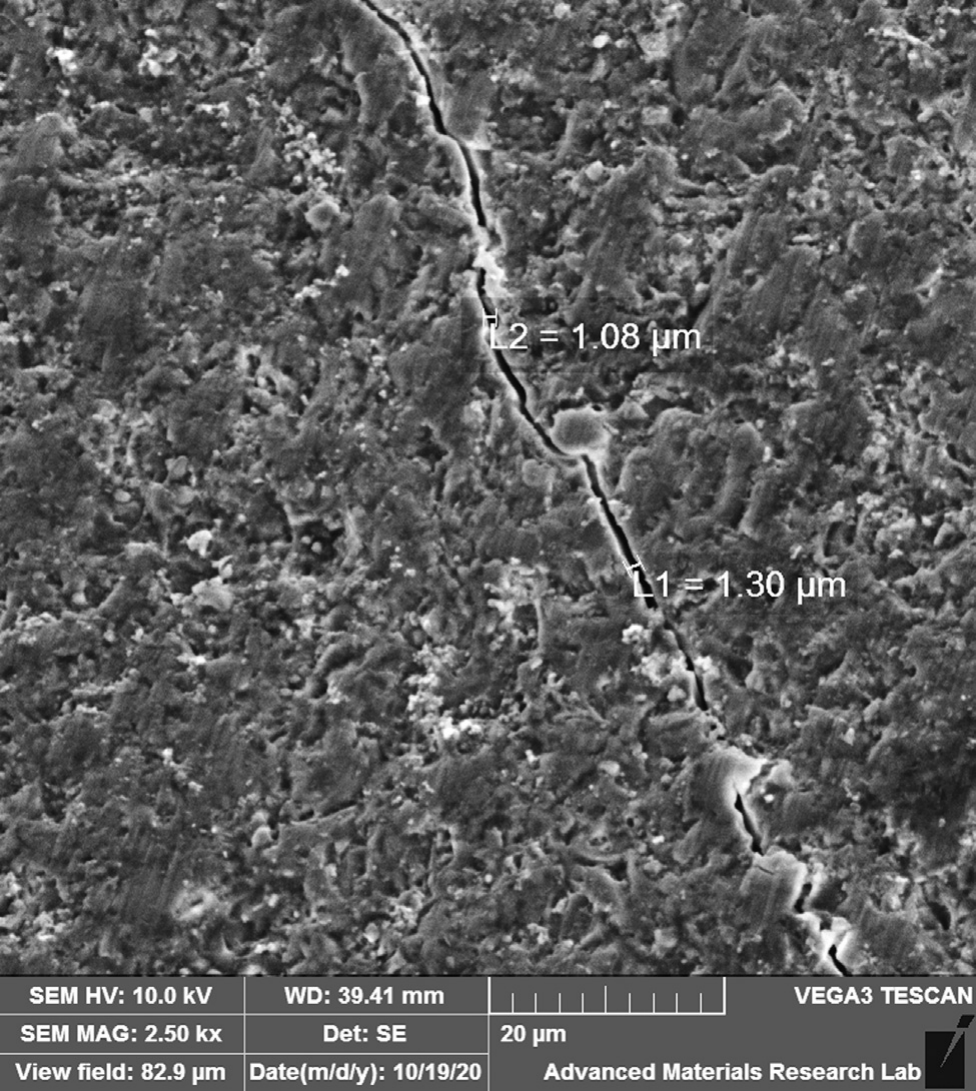
A scanning electron microscopic image (×2.5k magnification) showing a crack line in the ultra-thin laminate veneers following the thermomechanical fatigue.

**Fig. 3:**
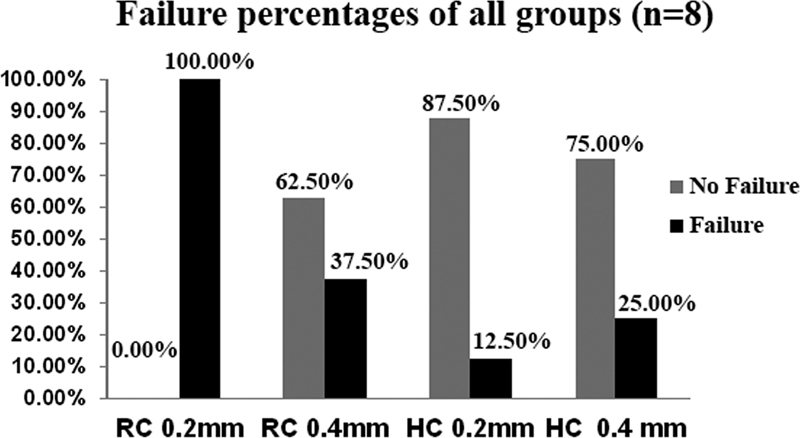
Fisher's exact probability test results of the fatigue resistance of the tested experimental groups. HC, resin composite; RC, resin cement.

Following the thermomechanical aging in the chewing simulator, the samples were examined under the SEM for fractures or cracks on the veneers ( Figs. 1 and 2 ). Regarding the Fatigue resistance test results, Fig. 3 highlights the results from Fisher's precise probability test. the proportions of failure of various groups are statistically significantly different ( *p* < 0.05). Cementation of 0.4-mm UTLV with RC demonstrated significantly higher fatigue resistance ( *p* < 0.05) than 0.2-mm UTLV; however, cementation with HC showed no significant difference between the two thicknesses tested ( *p* > 0.05) ( Table 2 ).

**Table 2:** Fisher's exact probability of the fatigue resistance test results

	**Fatigue failure**				** Statistical significance a**	**Total**
	**No failure**		**Failure**			
	**Count**	**%**	**Count**	**%**		
RC 0.2 mm	0	0.0	8	100.0	A	100.0%
RC 0.4 mm	5	62.5	3	37.5	B	100.0%
HC 0.2 mm	7	87.5	1	12.5	B	100.0%
HC 0.4 mm	6	75.0	2	25.0	B	100.0%

Abbreviations: HC, resin composite; RC, resin cement.

a Groups identified by the same letters are not significantly different ( *p* > 0.05). Different letters identify significantly different groups ( *p* < 0.05).

**Fig. 4:**
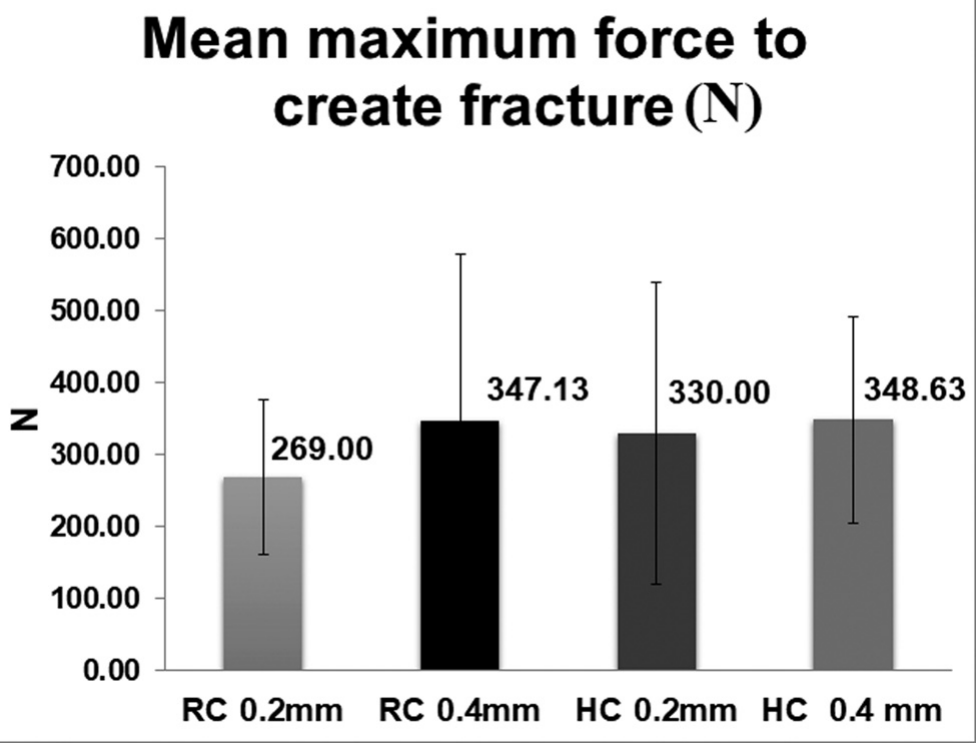
Mean maximum force values and standard deviation of the fracture resistance of the tested experimental groups. HC, resin composite; RC, resin cement.

**Fig. 5:**
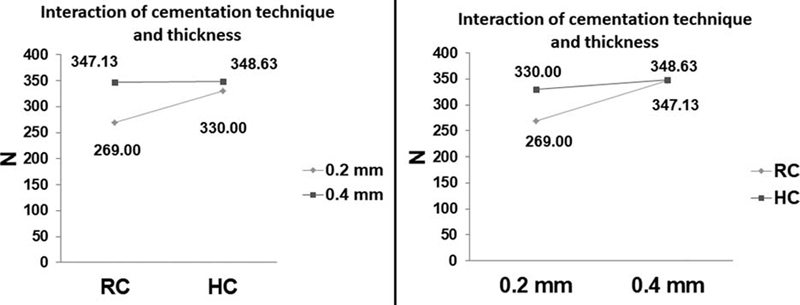
The interaction of the tested variable; cementation technique (RC and HC) and ultra-thin laminate veneers thickness (0.2 and 0.4 mm). HC, resin composite; RC, resin cement.

The fracture resistance test results were recorded in N, and the data passed the Shapiro–Wilk's test of normality, and a parametric test was applied to analyze the results. Descriptive statistics are listed in Table 3 and Fig. 4 . The interaction of bonding technique and veneer thickness using the GLM is illustrated in Fig. 5 . The results of the two-way ANOVA are listed in Table 4 , and show that both the two main effects and their interaction are statistically not significant ( *p* > 0.05). Furthermore, the estimated marginal means and pair-wise comparisons (Bonferroni adjustment) for bonding technique and thickness did not show statistical significant difference between the experimental groups ( *p* > 0.05).

**Table 3:** Descriptive statistics, mean and standard deviation of the fracture force (N) of the experimental groups.

	***N***	**Mean**	**SD**	**95% confidence interval for mean**	
				**Lower bound**	**Upper bound**
RC 0.2 mm	8	269.00	108.46	178.32	359.68
RC 0.4 mm	8	347.13	231.00	154.01	540.24
HC 0.2 mm	8	330.00	210.05	154.39	505.61
HC 0.4 mm	8	348.63	143.57	228.59	468.66
Total	32	323.69	174.38	260.82	386.56

Abbreviations: HC, resin composite; RC, resin cement; SD, standard deviation.

Interaction not significant ( *p* > 0.05). Main effect of veneer thickness and bonding technique was not significant ( *p* > 0.05).

**Table 4:** Analysis of variance of the fracture resistance test results

	**Sum of squares**	**df**	**Mean square**	***F***	***p*** **-Value**
Corrected model	33,614.13	3	11,204.71	0.35	0.79289
Intercept	3,352,755.13	1	3,352,755.13	103.27	0.00000
Bonding technique	7,812.50	1	7,812.50	0.24	0.62756
Thickness	18,721.13	1	18,721.13	0.58	0.45397
Bonding technique ^x^ thickness	7,080.50	1	7,080.50	0.22	0.64411
Error	909,008.75	28	32,464.60		
Total	4,295,378.00	32			
Corrected total	942,622.88	31			

## Discussion

Fracture is the main reason for the failure of laminate veneers, 19 and is responsible for 67% of the total failure rate recorded for porcelain veneers across 15 years of clinical service. 20 Considering how crucial veneers are for people seeking a smile change, the present study for fracture and fatigue resistance of the laminate veneers is of utmost importance. The cementation technique and thickness of the UTLV showed a statistically significant effect in fatigue resistance ( *p* < 0.05) but not in fracture resistance. In terms of fracture strength, the interaction of the two main effects, the bonding technique and the veneer thickness, was statistically nonsignificant ( *p* > 0.05). The experimentations show a statistically significant difference in fatigue resistance ( *p* < 0.05). The effect of thickness on fatigue resistance was observed between the groups, with 0.4 mm being statistically higher than the 0.2 mm veneer thickness when using the RC as a luting agent ( *p* < 0.05). There was, however, no statistical difference in the thickness of the veneer when using the preheated HC restoration ( *p* > 0.05). The bonding technique's impact on the results, with preheated HC restorations, showed a statistical difference from RC in veneer thicknesses of 0.2 mm ( *p* < 0.05), but no difference was found in cement type with veneer thicknesses of 0.4 mm ( *p* > 0.05). However, the statistical analysis does showed a nonsignificant difference in fracture loads between the groups ( *p* > 0.05), with the highest mean Recorded for the HC-0.4 group. In contrast, the fatigue resistance showed a significant difference between the groups. Based on the results from the study, the hypothesis stating that the thickness and bonding techniques have no effect on the fatigue and fracture resistance of UTLV can be partially rejected. The second null hypothesis stating that the two testing approaches used in this study would not deliver different results should also be rejected, as one test showed significant differences between the test groups.

The minimally invasive UTLV technique is a new approach of tooth preparation, limiting the preparation to a depth of 0.2 to 0.3mm into the enamel surface, which provides higher bond strength and fracture resistance. 7 . Increased porcelain thickness substantially raised the loads to failure on enamel substrates, while high porcelain thickness only moderately raised the loads to loss on all-dentin or half-enamel-half-dentin substrates 2 The difference in fracture resistance of UTLV when bonded to enamel or dentin is related to the difference between the modulus of elasticity (MOE) of the porcelain and that of the enamel and dentin. The MOE of porcelain is 70 GPa, which is very close to that of the enamel (84 GPa) and much higher than that of dentin (19 GPa). These findings explain the high failure rate of the veneers when bonded to dentin, as the dentin substrate cannot provide the necessary support of the brittle porcelain veneers. 21 According to the findings of this investigation, the thickness of veneers and the bonding technique have a statistically significant effect on the fatigue failure after thermomechanical cycling. The present study results showed that the fatigue resistance of the veneers with 0.2 mm thickness is comparable to that of 0.4-mm-thick veneers if they were cemented with preheated HC restoration.

Under compressive load, glass ceramics are known for their resilience, but they are brittle under tensile stress or intense pressure. Brittle fractures usually occur upon failure with minimal plastic deformation of the material's microstructure. 5 Cervical chipping is not directly caused by the impact of compressive bite force, since this failure is introduced by the tensile stresses formed at the veneers' underside. The effect of tensile force resistance may then be considered the reason behind the significant difference between the groups when testing the fatigue resistance in the chewing simulator, as the mechanical forces are a combination of shear, compressive, and tensile. The origin of the fracture is often near the location where the highest concentration of tensile stress builds up under bite load. In contrast, the force is only shear in the universal testing machine, indicating no significant difference between the groups. Microscopic defects are frequently the source of initial cracks, causing fracture when the pressure exceeds a critical level. 13

It was postulated that the porcelain failure originates from their cementation (intaglio) surface and that it may be a result of the tensile stresses within the ceramic at its interface with the luting agent, which occur from a minor bending of the ceramic under chewing loads on occlusal wear facets. 22 The typical bite force of the human teeth ranges between 20 and 1,000 N, while during actual chewing, the force does not exceed 270 N. Furthermore, the mean force in the anterior area of the mouth is less than in the posterior region, varying between 155 and 200 N. 23 The mean force of fracture resistance values of the tested groups were higher than 200 N, indicating that the tested veneer's thicknesses and bonding techniques can be acceptable clinically. To obtain more clinically acceptable data, a force of 40 N was applied to the anterior teeth for a total of 750,000 cycles, which was reported to simulate clinical aging for 5 years. 24

The direction of force is also playing a role in force of fracture, as the force in the universal testing machine is shear as in the studies of veneers if it is in the incisal edge and perpendicular to the tooth or veneers with different angles, as reported by Troedson and Dérand as maximum stresses increased four times when the load angle was 30° as compared with 0° and 1.5 times from 30° to 60° 21. The present is at 30° as the force directed perpendicular to the veneers; this may explain no significant difference between the groups.

Cementation of laminate porcelain, which is affected by the luting agent, gives significant support to the strength of the veneers. Luting the laminate veneer to the tooth via conventional RC is the most popular protocol for cementation today. RC based on bisphenol A-glycidyl methacrylate resin was introduced to the profession in the late 1970s, 15 which was found to increase the fracture resistance of porcelain laminate veneers. 17 Over the years, scholarly attention in the adhesive cementation of the indirectly fabricated restorations shifted toward the use of highly filled HC restorations. A less viscous material can be obtained by preheating the restorative material in a specific device without altering the properties. 25

Luting the veneer to the tooth via preheated HC restoration, which may provide superior mechanical and esthetic properties than conventional RC. 3 The warming devices of the HC restoration reduce the viscosity of the material, enhance the handling features, and improve the adaptability of these materials. After the cementation and cooling of the material, it becomes easy to remove the extruded excess material, due to the firm consistency and limitless working time until light polymerization, making them superior to traditional RC. 12 At 25°C, the composites are up to 38 times more viscous than the RC, whereas at 69°C, the difference is five times. Accordingly, the strengthening effect for the preheated HC is most significant. 26 The Calset Composite Warmer (AdDent Inc, Danbury, Connecticut, United States) takes 20 minutes to reach the optimum temperatures (54–68°C) that have been considered in the literature to adequately preheat the HC restoration, and then another 3 to 4 minutes to warm the material. 10 In a recent study by Marcondes et al, 10 preheated restorative HCs showed a rapid reduction in the temperature following preheating, and IPS Empress Direct (nanohybrid), which were used in the current study, exhibited the best results. The author stated that slow cooling increases the working time and maintain the low viscosity of the HC, and thus, the low film thickness of the material and that particle type, shape, size, nature of particle surface, and filler spatial arrangement within the resin composite may influence the viscosity and film thickness rather than filler contents. 27

The results of the current study confirmed that luting the veneers using preheated HC restoration would be a more valuable option, specifically with the minimal invasive preparation for 0.2 mm veneer thickness. Our fatigue resistance results are in agreement with those reported by Gresnig, 17 who examined the effect of luting agents on the load to failure value and accelerated fatigue resistance of lithium disilicate laminate veneers after thermomechanical aging. Another contributing factor to the high fatigue resistance of the samples bonded with HC, is the high filler loading of the restorative resin composite (79&), in comparison to <38& filler loading for RC. The study also tested the same groups in the universal testing machine and revealed a significantly higher fracture resistance of the preheated HC restoration than conventional RC. 17 Perhaps the discrepancy with the present study results is due to the material used and the difference in the preparation design which may have influenced the results.

It seems that many factors play a role in determining the success and survival of laminate veneers. These factors depend more on the clinical technique than the laboratory procedure and even more on the cementation method. Nevertheless, high success of veneers has been reported in the literature.13 According to Layton and Walton, the average survival rate for feldspathic porcelain veneers bonded to prepared enamel was 96 percent after 21 years. 28 Another study by the same authors examined the results of 304 feldspathic porcelain veneers prepared by the same dentist for 100 patients over 16 years; the preparations were done with a palatal overlap design, and 80% of the preparations were in enamel. The authors found that the survival rate for veneers was 91% at 12 to 13 years and 73% at 16 years. 29 Integrating the effect of veneer thickness and the type of luting agent, feldspathic porcelain veneers with a thickness of 0.3 mm or less and adhesive RC provided the required fracture resistance. 29 , 30 However, in our study, there was a difference in the interaction between the veneer thickness and bonding technique in terms of fracture resistance, but it was not statistically significant. The difference in results may be due to different sample preparation or different ceramics tested.

## Conclusion

Within the limitations of this study, the findings of this study support bonding with preheated HC when luting UTLV with a thickness less than 0.4 mm. Preheated HCs were found to increase the fatigue resistance of the UTLV in comparison to the conventional RC.
